# Thrombomodulin and Thrombopoietin, Two Biomarkers of Hemostasis, Are Positively Associated with Adherence to the World Cancer Research Fund/American Institute for Cancer Research Recommendations for Cancer Prevention in a Population-Based Cross-Sectional Study

**DOI:** 10.3390/nu11092067

**Published:** 2019-09-03

**Authors:** Mirja Grafetstätter, Laura Pletsch-Borba, Disorn Sookthai, Nena Karavasiloglou, Theron Johnson, Verena A. Katzke, Michael Hoffmeister, Peter Bugert, Rudolf Kaaks, Tilman Kühn

**Affiliations:** 1 Division of Cancer Epidemiology, German Cancer Research Center (DKFZ), Im Neuenheimer Feld 581, 69120 Heidelberg, Germany; 2 Division of Chronic Disease Epidemiology, Institute for Epidemiology, Biostatistics and Prevention, University of Zürich, 8001 Zürich, Switzerland; 3 Division of Clinical Epidemiology and Aging Research, German Cancer Research Center (DKFZ), Im Neuenheimer Feld 581, 69120 Heidelberg, Germany; 4 Institute of Transfusion Medicine and Immunology, Medical Faculty Mannheim, and German Red Cross Blood Service, Friedrich-Ebert-Str. 107, 68167 Mannheim, Germany

**Keywords:** lifestyle, coagulation, thrombomodulin, thrombopoietin, fibrinogen, cancer prevention, WCRF/AICR recommendations

## Abstract

A pro-coagulative state is related to increased risk of cardiovascular diseases but also certain cancers. Since experimental and smaller human studies suggest that diet, physical activity, and body weight may all affect coagulation, we evaluated associations between these lifestyle factors and hemostatic biomarkers in a population-based study. Cross-sectional baseline data from 2267 randomly selected participants of EPIC-Heidelberg (age range 35–65 years) was used. Fibrinogen, glycoprotein IIb/IIIa, P-selectin, thrombomodulin (TM), and thrombopoietin (TPO) were measured in baseline plasma samples. A score reflecting adherence to the World Cancer Research Fund/American Institute for Cancer Research (WCRF/AICR) recommendations for cancer prevention was created. Associations between the WCRF/AICR score as well as its individual components and hemostatic biomarkers were analyzed by linear regression models. Multivariable-adjusted geometric means (95% confidence intervals) of TM and TPO were higher with greater adherence to the WCRF/AICR recommendations (TM, lowest vs. highest score category: 2.90 (2.7,3.1) vs. 3.10 (2.9,3.3) ng/mL, *p*_linear trend_ = 0.0001; TPO: 328 (302,356) vs. 348 (321,378) pg/mL, *p*_linear trend_ = 0.0007). These associations were driven by lower alcohol and meat consumption among persons with higher WCRF/AICR scores. Our results indicate that lifestyle factors favorably affect TM and TPO, two hemostatic factors implicated in chronic disease development.

## 1. Introduction

Beyond its established role as an important risk factor for cardiovascular diseases, a pro-coagulative state, as reflected by increased platelet activation and elevated plasma level of coagulation factors, is linked to carcinogenesis [1,2,3,4] and poor prognosis among cancer patients [5]. An overall health-conscious lifestyle may help preventing increased coagulation and its cardiovascular complications [6], and we and others have previously shown that smoking may be a strong driver of coagulation and platelet activation [1]. However, to our knowledge, a comprehensive evaluation of nutrition-related lifestyle factors (i.e., food intake, body weight, and physical activity) in relation to hemostatic biomarkers from population-based human studies is missing.

Based on systematic reviews, the World Cancer Research Fund and the American Institute for Cancer Research (WCRF/AICR) has published 10 global cancer prevention recommendations focusing on dietary factors, physical activity, and weight management (see Table 1) [7]. In order to assess whether adherence to these lifestyle recommendations has an impact on parameters of coagulation and platelet activation beyond known determinants, as particularly smoking, we investigated associations between hemostatic biomarkers and adherence to the WCRF/AICR recommendations using a questionnaire-based score [8] among a random subcohort (*n* = 2267) of the prospective European Investigation into Cancer and Nutrition (EPIC)-Heidelberg. The score and its individual components were analyzed in relation to five biomarkers of hemostasis, glycoprotein (GP) IIb/IIIa, P-Selectin, thrombopoietin (TPO), thrombomodulin (TM), and fibrinogen, which were selected based on literature review [1,9]. In short, GPIIb/IIIa and P-Selectin are both platelet integral membrane proteins, which are expressed on the platelet surface upon platelet activation [10], and play a crucial role in platelet aggregation and adhesion. TPO constitutes the main physiological regulator of platelets as it stimulates the growth of megakaryocytes, i.e., platelet progenitor cells [11]. Binding of TPO to its receptor on the platelet surface results in TPO internalization and degradation which explains that levels of the molecule have been reported to be inversely proportional to platelet rates [11]. TM is a transmembrane protein with anti-coagulative properties [12]. In addition, we selected the plasmatic coagulation factor fibrinogen, which has an important function in platelet activation and aggregation and is needed for clot stabilization [13].

## 2. Materials and Methods

### 2.1. Study Population

Detailed recruitment and characteristics of the EPIC-Heidelberg study have been described previously [14]. In brief, EPIC-Heidelberg is part of the European EPIC project, a large population-based cohort study that was set up in the 1990s with the aim to investigate diet, lifestyle, genetic, and environmental factors in relation to cancer and other chronic diseases. Overall, 25,540 individuals (53.3% women) aged 35–65 were recruited from the local general population for EPIC-Heidelberg between 1994 and 1998. For the present study, data of a random subcohort (*n* = 2480) were analyzed, which comprised about 10% of all EPIC-Heidelberg participants and showed highly similar characteristics compared to the full cohort [9]. Participants with missing data on variables needed for the WCRF/AICR score (body weight *n* = 15, and breastfeeding *n* = 14) were excluded from the analyses. Furthermore, we excluded participants with missing data on the following covariables: smoking status (*n* = 7), low-density lipoprotein (LDL) (*n* = 159), and C-reactive protein (CRP) (*n* = 18). Finally, 2267 participants (*n* = 1196 women and *n* = 1071 men) were included into the analyses.

The study was approved by the Ethics Committee of the Heidelberg University Hospital (Heidelberg, Germany) and all participants gave written consent for the use of their data and blood samples. The study was performed in accordance with the Declaration of Helsinki.

### 2.2. Data Collection

Detailed lifestyle data including information on smoking, alcohol consumption, physical activity, education, reproductive history, use of exogenous hormones, and breastfeeding was obtained from standardized self-administered questionnaires and PC guided interviews at the initial study examinations. To assess habitual dietary intake, a validated, self-administered 158-item food frequency questionnaire was used [15], in which participants were asked to report their average frequency of consumption over the previous 12 months. Furthermore, anthropometric measurements were carried out and blood samples were taken by trained personnel according to standardized procedures [16].

### 2.3. Laboratory Methods

At baseline, blood was processed into serum, plasma, buffy coat, and erythrocyte samples and prepared for long-term storage in gas phase liquid nitrogen (−150 °C). For analyses of routine blood parameters as covariates—including concentrations of high-density lipoproteins (HDL), triglycerides (TG), total cholesterol (TC), and CRP—serum samples were sent on dry ice to the Scandinavian Health Ltd. Laboratories in the Netherlands, where they were measured using the Roche Cobas 6000 analytical system, according to manufacturer’s protocols. Levels for LDL were calculated with the Friedewald Formula (LDL = TC-HDL-TG/5) [17]. For analyses of hemostatic biomarkers, plasma aliquots were thawed for the first time in 2016. Electrochemiluminescence immunoassays (ECLIA) were carried on the Quickplex SQ 120 instrument (Meso Scale Discoveries, MSD, Maryland, USA) to measure plasma concentrations of P-selectin, TM, and TPO, using MSD’s ‘human vascular injury I’ (P-selectin and TM) and ‘U-Plex TPO assay’ (TPO) kits. GPIIb/IIIa and fibrinogen levels were measured by enzyme-linked immunosorbent assays (ELISA) using the assay kits ‘ab108851’ from Abcam (Cambridge, UK) and ‘KA0475’ from Abnova (Heidelberg, Germany). Each analytical batch contained two quality control (QC) plasma samples in duplicate in order to monitor the validity of the measurements within and across batches. Within-batch coefficients of variation (CVs) (between-batch CVs) were 4.6% (19.5%), 3.8% (10.1%), 3.3% (9.1%), 5.5% (46.9%), and 5.7% (8.5%) for TPO, TM, P-selectin, GPIIb/IIIa, and fibrinogen. For all markers the percentages of missing values were below 1% (TPO: *n* = 1, TM: *n* = 3, P-selectin: *n* = 2, fibrinogen: *n* = 1, and GPIIb/IIIa: *n* = 23).

### 2.4. WCRF/AICR Score

As described earlier by Romaguera et al. [8], we constructed a score, incorporating eight of the ten recommendations for cancer prevention (body weight, physical activity, the consumption of plant-based foods, energy-dense foods, sugary drinks, alcohol, red and processed meat, and breastfeeding for women), which have been published in the updated WCRF/AICR report in 2018 [7]. Due to the lack of information about the purpose, exact durations and dosages of use, we did not include the recommendation on supplement intake. As the WCRF/AICR special recommendation for cancer survivors was not applicable to our study population, this recommendation was neither included.

Details on the score construction and cut-offs used are shown in Table 1. When the recommendation was fully met, we assigned 1 point for the component, when it was partially met 0.5 points, otherwise 0 points. For the recommendation on plant foods, which includes two sub-recommendations, the final score was the average of both sub-recommendations. All recommendations were summed and contributed equally to the WCRF/AICR score which ranged from 0 to 7 in men and from 0 to 8 in women (since the recommendation on breastfeeding is applicable only to women), with higher scores indicating greater adherence to the WCRF/AICR recommendations. Based on its distribution in the study population, the score was categorized as follows: category 1 (≥0 to ≤2 in men; ≥0 to ≤3 in women), category 2 (>2 to ≤3 in men; >3 to ≤ 4 in women), category 3 (>3 to ≤4 in men; >4 to ≤5 in women), and category 4 (>4 in men; >5 in women).

### 2.5. Statistical Analyses

Descriptive characteristics of the study population are reported as percentages for categorical variables or means and standard deviations for continuous variables.

Generalized linear models were used to assess associations between biomarker levels and the WCRF/AICR score, which was included into the model as a categorical variable (using the four categories). Linear trend tests were tested modelling the categorical score as a continuous parameter. Two models with different levels of adjustment were used. The first model (Model 1) only included age at recruitment (in years) and sex (male, female) as potential confounders. Model 2 further comprised smoking status (never smoked, quit ≥10 years ago, quit <10 years ago, smoked < 15 cigarettes per day at baseline, smoked ≥ 15 cigarettes per day at baseline), education level (primary school, secondary school, university degree), current aspirin use (yes/no), CRP levels (mg/dl, continuous), LDL levels (mg/dL, continuous, calculated with the Friedewald formula), total energy intake (KJ/day) as well as prevalent cancer (yes(no), prevalent myocardial infarction (yes/no) and prevalent stroke (yes/no). Analyses in women were further adjusted for menopausal status (pre-, peri-, postmenopausal), use of hormone replacement therapy (yes/no), use of contraceptive pills (yes/no) and for parity (at least one full term pregnancy, yes/no). To assess the associations between biomarker levels and individual score components, regression models adjusted for all other components of the score as well as the covariates used in Model 2 (as reported above) were used. Given the slightly different WCRF/AICR recommendations for women and men (i.e., the inclusion of breastfeeding into the lifestyle score among women), we decided to stratify all analyses by sex in subgroup analyses. This decision was further motivated by the observation of significant statistical interactions between the WCRF/AICR score (continuous) and sex in relation to some of the markers (TPO and TM).

All statistical analyses were performed with SAS Software (version 9.4; SAS Institute Inc., Cary, NC, USA).

## 3. Results

An overview of the WCRF/AICR recommendations and the score construction as well as adherence to the recommendations within EPIC Heidelberg is shown in Table 1. The recommendation most participants adhered to was limiting the consumption of alcohol to one drink per day (58.5% of the participants). The physical activity recommendation was met by 52.0% of the participants and 44.6% individuals were within the recommended BMI range, while less than 10% of the participants fully met the recommendations on energy density, sugary drinks, and red and processed meat intake.

Baseline characteristics of the study population according to categories of the WCRF/AICR score are depicted in Table 2. Participants with greater adherence to the WCRF/AICR recommendations were more highly educated and less likely to smoke.

**Figure 1:**
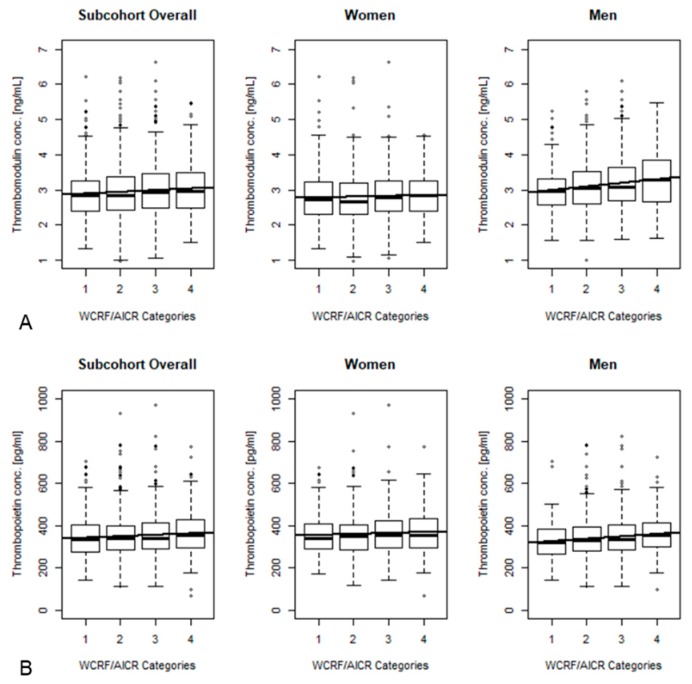
Thrombomodulin (**A**) and thrombopoietin (**B**) concentrations across WCRF/AICR score categories (*n* = 2276, 1196 women and 1071 men).

In the age- and sex-adjusted linear regression model, levels of fibrinogen were significantly inversely associated with the WCRF/AICR sore, while levels of TM as well as TPO showed significant positive associations (Supplementary Table S1). Upon multivariable adjustment, the association between WCRF/AICR score and fibrinogen was attenuated and no longer statistically significant (Table 3), while the positive associations with TM and TPO remained significant. When stratifying the analyses by sex, these associations were slightly stronger with respect to TPO and TM among men (Figure 1 and Table 3). Additional adjustment for glucose levels, fasting status, and hemoglobin (Hb)A1c levels did not substantially affect the associations between WCRF/AICR and biomarker levels (Supplementary Table S2).

Analyses on individual components of the WCRF/AICR score and biomarker levels are shown in Table 4. While fibrinogen levels decreased with greater adherence to the recommendation on body weight, levels increased with lower alcohol consumption. The same pattern with respect to the recommendation on alcohol consumption was observed for TM and TPO, where levels increased with greater adherence the WCRF/AICR recommendations, i.e., lower consumption. Levels of TPO and TM were further positively associated with greater adherence to the recommendations on meat consumption. Additionally, we observed significantly higher levels of P-Selectin among individuals who consumed more energy-dense foods compared to individuals with a lower consumption and significantly higher levels of GPIIb/IIIa among individuals with a high consumption of sugar sweetened drinks compared to those with no consumption.

Sex-stratified analyses on the association between individual components of the WCRF/AICR score and biomarker levels are outlined in Supplementary Tables S3 and S4. Overall, there were only slight differences in the associations between score components and biomarkers levels of women and men. Levels of TM and P-Selectin were lower among women, but not among men with greater adherence to the WCRF/AICR recommendation on body weight. Furthermore, fibrinogen levels were lower with increased physical activity among women, while GPIIb/IIIa levels were lower with higher consumption of sugar sweetened drinks among men.

## 4. Discussion

This cross-sectional, population-based study among middle-aged European women and men was the first study to investigate whether biomarkers of blood coagulation and hemostasis are influenced by healthy lifestyle habits as reflected by a composite WCRF/AICR lifestyle score. After comprehensive adjustment for confounders, we observed higher levels of TM and TPO in individuals with greater adherence to the WCRF/AICR score indicating a more favorable lifestyle pattern. When evaluating individual components of the lifestyle score, TM and TPO were inversely associated with the consumption of alcohol as well as red and processed meat. There was no significant association between either fibrinogen, GPIIb/IIIa, or P-Selectin and the composite WCRF/AICR lifestyle score.

### 4.1. Thrombomodulin Levels in Relation to Lifestyle and Chronic Diseases

TM, which showed a positive association with a more favorable lifestyle pattern in our study, has been associated with cardiometabolic diseases in several studies. While increased TM levels were associated with lower risks of type 2 diabetes mellitus in the prospective MONICA/KORA study [18], no association was found between TM and the risk of coronary heart disease (CHD) in the same population [19]. Within the prospective ARIC study on the contrary, an inverse association between TM and incident CHD was observed [20]. However, TM levels were positively associated with carotid atherosclerosis in cross-sectional analyses, also within the ARIC study [20] and have further been reported to be higher in patients with type 2 diabetes mellitus compared to healthy controls [21]. The authors discussed that circulating TM in healthy individuals may mainly reflect TM production on endothelial cells, indicating a low pro-thrombotic state and thus decreased diabetes and cardiovascular risk, while high TM levels in the plasma of patients may be caused by endothelial dysfunction and subsequent increase of TM by cell injury, which could in turn mask decreased TM production [18,20]. This hypothesis would be in line with our observation of increased TM level among individuals following a more favorable lifestyle. In addition to its relation to cardiometabolic diseases, TM has also been associated with cancer. While TM expression in tumor tissues is suggested to correlate with better prognosis [22], high levels of TM in plasma have been associated with cancer progression in a smaller study [23]. Then again, we have previously shown that pre-diagnostic plasma TM is not associated with risks of common cancers [1,9]. Overall, the evidence on TM and cancer is still limited.

### 4.2. Thrombopoietin Levels in Relation to Lifestyle and Chronic Diseases

To our knowledge, TPO has not been evaluated in relation to nutritional factors before. Considering that TPO levels may be inversely proportional to platelet counts [11], which in turn have been related to unhealthy lifestyle habits [24,25] and increased disease risks [26], our observation of high TPO concentrations among individuals, who mostly adhere to the cancer prevention recommendations appears plausible. On the other hand, TPO has also been reported to be stored in platelet granules and released upon platelet activation [27], explaining high TPO levels in patients suffering from disseminated intravascular coagulation, which is accompanied by massive platelet activation [27]. In patients with coronary artery disease [28] and cancer [29], elevated TPO levels have also been observed and it was suggested that higher TPO may contribute to disease progression. Although underlying mechanisms remain unclear, elevated TPO levels observed in patients could be a result of increased platelet damage [28], or tumor-derived thrombopoietic cytokine production [29]. It also possible that TPO is released into circulation from already activated platelets. By contrast, our observation of high TPO levels among more health-conscious individuals might reflect low platelet counts and a low thrombotic activity in the general population. In a previous study, we found that pre-diagnostic TPO levels were inversely associated with colon cancer risk among men, but not women [9], which is in agreement with the slightly stronger association between lifestyle and TPO levels among men in the present analysis. 

### 4.3. Alcohol Consumption and Biomarker Levels

Inverse associations between fibrinogen levels and alcohol consumption have been reported previously; however, results vary with regard to the reported amounts of alcohol intake. In the Framingham Offspring cohort, light to moderate alcohol consumption (defined as 7–21 drinks per week) was associated with lower levels of fibrinogen while heavy alcohol consumption (≥21 drinks per week) appeared to shift the direction towards higher fibrinogen levels [30]. In turn, moderate alcohol consumption (i.e., consuming ≤ 10–20 g alcohol per day) in our study, was related to higher levels of fibrinogen compared to individuals who consumed more than 20–30 g alcohol per day and would be consistent with a metal-analysis, in which lower fibrinogen levels were associated with the consumption of 30 g alcohol per day [31]. While lower alcohol consumption may thus be associated with higher fibrinogen, an established cardiovascular risk factor [32,33], it was also associated with higher TPO and TM levels, which may reflect lower cardio-metabolic risks, in our study (see above). We can only speculate that potential different effects of alcohol consumption on hemostatic factors may underlie differential associations between alcohol consumption and different cardiovascular endpoints [34].

### 4.4. Strengths and Limitations

Strengths of this study include the population-based set-up, the large sample size, the well-characterized cohort, the meticulous statistical adjustment, and the comprehensive set of biomarkers. At the same time, the WCRF/AICR lifestyle score is limited in that each underlying recommendation is equally weighted, while the associations between individual dietary items and cancer risks are often weaker than those between BMI and cancer risk. It should be noted that the score does not include smoking, a lifestyle factor essential for the prevention of chronic diseases, for which we and others have shown associations with biomarkers of a pro-coagulative state, especially P-selectin and fibrinogen [1,35,36]. We cannot rule out that some of our subgroup findings were due to chance, given the high number of analyses. However, our results on individual score items and fibrinogen, the only routine biomarker in our study, are consistent with those of previous studies, suggesting that EPIC-Heidelberg is a valid model for analyses on lifestyle and hemostatic factors. Nevertheless, considering that TPO and TM are still subject to basic research and reference ranges for optimal plasma levels in different populations do not exist, the present findings of associations with lifestyle factors should be interpreted with caution, and intervention trials are needed to further investigate potential effects of lifestyle modification on these and other biomarkers of hemostasis. In this context, an additional assessment of hemostatic routine parameters such as platelet count, platelet volume, and platelet distribution width in relation to lifestyle factors, which was not possible in our study due to the lack of intact platelets, would be useful in future studies.

## 5. Conclusions

In summary, we observed higher plasma levels of TM and TPO among individuals with a more favorable lifestyle pattern. In particular, both markers were inversely associated with red and processed meat, and alcohol consumption. These observations are consistent with the hypothesis that lifestyle factors affect hemostasis, and that a pro-coagulative state may be amenable to lifestyle modification. The latter, however, needs to be proven by intervention trials.

## Figures and Tables

**Table 1:** Adherence to WCRF/AICR recommendations for cancer prevention in the EPIC Heidelberg subcohort and operationalization of the WCRF/AICR sore.

WCRF/AICR Recommendations	Baseline Data	Categories	Scoring	All (%)	Women (%)	Men (%)
Be a healthy weight	Body mass index (kg/m^2^)	18.5–24.9	1	44.6	54.7	33.2
		25–29.9	0.5	39.0	29.5	49.6
		<18.5 or ≥30	0	16.5	15.8	17.2
Be physically active	Physical activity level ^1^	High	1	52.0	50.6	53.6
		Medium	0.5	19.7	19.8	19.6
		Low	0	28.3	29.6	26.8
Limit energy dense foods consumption	Energy density (kcal*100 g^−1^*day^−1^)	≤125	1	7.5	10.6	3.9
		>125 to <175	0.5	58.4	61.5	54.8
		≥175	0	34.2	27.8	41.3
Limit sugar sweetened drinks consumption	Sweet drinks consumption (g/day)	0	1	4.4	4.0	4.7
		≤250	0.5	62.1	65.6	58.2
		>250	0	33.5	30.4	37.1
Eat a diet rich in whole grains, vegetables, fruits, and beans	Fruit and vegetable intake (g/day)	≥400	1	24.5	28.9	19.6
		200 to <400	0.5	71.5	67.6	75.9
		<200	0	4.0	3.6	4.5
	Dietary fiber intake (g/day)	≥ 25	1	19.9	16.0	24.4
		12.5 to <25	0.5	69.6	71.2	67.8
		< 12.5	0	10.5	12.8	7.8
Limit consumption of red and processed (RP) meat	Total RP meat intake (g/wk) and processed meat (P) intake (g/day)	<500 RP and <3 P	1	3.1	4.0	2.2
		<500 RP and 3 to <50 P	0.5	30.5	50.7	8.0
		≥500 RP and ≥50 P	0	66.3	45.3	89.8
Limit alcohol consumption	Ehanol intake (g/day) for men (m) and women (w)	≤20 (m), ≤10 (w)	1	58.5	65.2	51.0
		>20–30 (m), >1–20 (w)	0.5	17.2	17.1	17.3
		>30 (m), >20 (w)	0	24.4	17.7	31.8
Do not use supplements for cancer prevention	Not applicable to this population	NA	NA	NA	NA	NA
Special Recommendations						
For mothers: breastfeed your baby, if you can	Cumulative breastfeeding (in months)	≥6	1		25.8	
		>0 to 6	0.5		41.6	
		No breastfeeding	0		32.6	
For cancer survivors: follow the WCRF/AICR recommendations	Not applicable to this population	NA	NA	NA	NA	NA
WCRF/AICR categories	Categorization of scores (for men/for women)	0 to ≤2/0 to ≤3		16.5	18.0	14.8
		>2 to ≤3/>3 to ≤4		35.5	32.1	39.3
		>3 to ≤4/>4 to ≤5		33.0	31.9	34.3
		>4/>5		15.0	18.1	11.7

^1^ ‘high’ if reported heavy manual job or >2 h/wk vigorous physical activity or >30 min/day cycling/sports, ‘moderate’ if reported 15–30 min/day cycling/sports and ‘low’ if reported <15 min/day cycling/sports; EPIC = European Prospective Investigation into Cancer and Nutrition; WCRF/AICR = World Cancer Research Fund/American Institute for Cancer Research; g = gram; wk = week; m = men, w = women; NA = not available.

**Table 2:** Baseline characteristics of 2267 individuals of the EPIC Heidelberg subcohort according to categories of the WCRF/AICR score ^1^ by sex.

	WCRF/AICR Score Categories				
	All	Category 1	Category 2	Category 3	Category 4
**Women**					
Score range	0–8	0 to ≤3	>3 to ≤4	>4 to ≤5	>5
*n*	1196 (100)	215 (18.0)	384 (32.1)	381 (31.9)	216 (18.1)
Age at recruitment (y)	49.4 ± 8.7	49.5 ± 8.5	49.3 ± 8.8	49.8 ± 8.8	48.7 ± 8.4
Smoking status					
Never smokers	610 (51.0)	99 (46.0)	197 (51.3)	203 (53.3)	111 (51.4)
Long-term quitters (≥10 y)	213 (17.8)	39 (18.1)	55 (14.3)	69 (18.1)	50 (23.1)
Short-term quitters (<10 y)	125 (10.5)	19 (8.8)	42 (10.9)	43 (11.3)	21 (9.7)
Light smokers (<15 cig/day)	157 (13.1)	32 (14.9)	51 (13.3)	48 (12.6)	26 (12.0)
Heavy smokers (≥15 cig/day)	91 (7.6)	26 (12.1)	39 (10.2)	18 (4.7)	8 (3.7)
Education level					
Primary school	325 (27.2)	68 (31.6)	115 (29.9)	106 (27.8)	36 (16.7)
Secondary school	582 (48.7)	97 (45.1)	191 (49.7)	191 (50.1)	103 (47.7)
University degree	289 (24.2)	50 (23.3)	78 (20.3)	84 (22.0)	77 (35.6)
Aspirin use	37 (3.1)	7 (3.3)	12 (3.1)	14 (3.7)	4 (1.9)
Contraceptive pill use ^2^	67 (9.4)	12 (9.5)	20 (8.3)	21 (9.9)	14 (10.3)
HRT use ^3^	299 (45.4)	60 (48.8)	95 (43.8)	96 (44.4)	48 (47.1)
≥1 full term pregnancy	971 (81.2)	143 (66.5)	303 (78.9)	329 (86.4)	196 (90.7)
Menopausal status					
Premenopausal	538 (45.0)	92 (42.8)	167 (43.5)	165 (43.3)	114 (52.8)
Perimenopausal	176 (14.7)	34 (15.8)	73 (19.0)	47 (12.3)	22 (10.2)
Postmenopausal	482 (40.3)	89 (41.4)	144 (37.5)	169 (44.4)	80 (37.0)
**Men**					
Score range	0–7	0 to ≤2	>2 to ≤3	>3 to ≤4	>4
*n*	1071 (100)	158 (14.8)	421 (39.3)	367 (34.3)	125 (11.7)
Age at recruitment (y)	52.5 ± 7.2	52.4 ± 7.0	52.7 ± 7.1	52.2 ± 7.4	52.4 ± 7.1
Smoking status					
Never smokers	367 (34.3)	40 (25.3)	127 (30.2)	140 (38.1)	60 (48.0)
Long-term quitters (≥10 y)	300 (28.0)	49 (31.0)	119 (28.3)	101 (27.5)	31 (24.8)
Short-term quitters (<10 y)	125 (11.7)	20 (12.7)	48 (11.4)	43 (11.7)	14 (11.2)
Light smokers (<15 cig/day)	134 (12.5)	18 (11.4)	60 (14.3)	45 (12.3)	11 (8.8)
Heavy smokers (≥15 cig/day)	145 (13.5)	31 (19.6)	67 (15.9)	38 (10.4)	9 (7.2)
Education level					
Primary school	314 (29.3)	50 (31.6)	128 (30.4)	112 (30.5)	24 (19.2)
Secondary school	350 (32.7)	58 (36.7)	148 (35.2)	110 (30.0)	34 (27.2)
University degree	407 (38.0)	50 (31.6)	145 (34.4)	145 (39.5)	67 (53.6)
Aspirin use	66 (6.2)	10 (6.3)	23 (5.5)	23 (6.3)	10 (8.0)

Values are means ± standard deviations or proportions; ^1^ score adapted from Romaguera et al. [8]; ^2^ among pre- and perimenopausal women (*n* = 714); ^3^ among peri-and postmenopausal women (*n* = 658); EPIC = European Prospective Investigation into Cancer and Nutrition; WCRF/AICR = World Cancer Research Fund/American Institute for Cancer Research; y = years; cig = cigarettes; HRT = hormone replacement therapy.

**Table 3:** Geometric means (95% confidence intervals) of biomarker levels across WCRF/AICR categories within the EPIC-Heidelberg subcohort by sex.

	Subcohort	WCRF/AICR Score Categories				
		1	2	3	4	*P*_trend_
**All**						
*n* (%)	2267 (100)	373 (17)	805 (35)	748 (33)	341 (15)	
Score range		0 to ≤2/0 to ≤3	2 to ≤3/3 to ≤4	3 to ≤4/4 to ≤5	>4/>5	
Fibrinogen (mg/mL)	3.99 (3.81,4.18)	3.98 (3.79,4.18)	4.02 (3.83,4.21)	3.97 (3.78,4.16)	3.95 (3.76,4.15)	0.25
Glycoprotein IIb/IIIa (ng/mL)	402 (367,441)	408 (370.0,450.6)	402 (366,441)	401 (365,441)	402 (364,443)	0.51
P-Selectin (ng/mL)	27.2 (24.6,30.0)	27.5 (24.7,30.6)	26.8 (24.2,29.7)	27.3 (24.7,30.3)	27.5 (24.8,30.6)	0.67
Thrombomodulin (ng/mL)	2.98 (2.8,3.2)	2.90 (2.7,3.1)	2.93 (2.7,3.1)	3.00 (2.8,3.2)	3.10 (2.9,3.3)	0.0001
Thrombopoietin (pg/mL)	337 (312,364)	328 (302,356)	331 (306,358)	341 (315,370)	348 (321,378)	0.0007
**Men**						
*n* (%)	1071 (100)	158 (15)	421 (39)	367 (34)	125 (12)	
Score range		0 to ≤2	2 to ≤3	3 to ≤4	>4	
Fibrinogen (mg/mL)	3.94 (3.76,4.13)	4.06 (3.80,4.33)	4.03 (3.79,4.28)	3.99 (3.75,4.24)	4.03 (3.78,4.30)	0.50
Glycoprotein IIb/IIIa (ng/mL)	399 (364,438)	391 (343,444)	393 (349,444)	381 (337,430)	372 (327,424)	0.12
P-Selectin (ng/mL)	28.9 (26.2,32.0)	30.5 (26.3,35.3)	29.52 (25.8,33.8)	30.2 (26.3,34.7)	32.1 (27.7,37.1)	0.24
Thrombomodulin (ng/mL)	3.13 (2.9,3.3)	2.93 (2.7,3.2)	3.03 (2.8,3.3)	3.10 (2.9,3.4)	3.28 (3.0,3.6)	0.0001
Thrombopoietin (pg/mL)	327 (302,354)	301 (271,335)	314 (285,346)	324 (293,358)	343 (309,381)	0.0001
**Women**						
*n* (%)	1196 (100)	215 (18)	384 (32)	381 (32)	216 (18)	
Score range		0 to ≤3	3 to ≤4	4 to ≤5	>5	
Fibrinogen (mg/mL)	4.04 (3.85,4.23)	3.76 (3.40,4.16)	3.85 (3.49,4.25)	3.76 (3.41,4.15)	3.76 (3.39,4.16)	0.51
GPIIb/IIIa (ng/mL)	405 (369,446)	485 (394,596)	468 (382,573)	481 (392,590)	495 (402,611)	0.41
P-Selectin (ng/mL)	25.5 (23.0,28.3)	21.1 (17.1,25.9)	20.2 (16.5,24.8)	21.0 (17.1,25.7)	20.6 (16.7,25.4)	0.87
Thrombomodulin (ng/mL)	2.84 (2.7,3.0)	2.69 (2.3,3.1)	2.66 (2.3,3.1)	2.76 (2.4,3.2)	2.83 (2.4,3.3)	0.020
Thrombopoietin (pg/mL)	347 (320,376)	350 (294,417)	340 (287,404)	355 (299,422)	366 (307,437)	0.058

Linear regression model adjusted for age, sex (where applicable), education, smoking status, aspirin intake, energy intake, CRP levels, LDL levels, prevalent cases of cancer, prevalent cases of myocardial infarction and stroke, women only: menopausal status, use of hormone replacement therapy, use of contraceptive pills, full term pregnancy; EPIC = European Prospective Investigation into Cancer and Nutrition; WCRF/AICR = World Cancer Research Fund/American Institute for Cancer Research.

**Table 4:** Geometric means (95% confidence intervals) of biomarker levels across individual components of the WCRF/AICR recommendations within the EPIC-Heidelberg subcohort (*n* = 2267).

Score	*n* (%)	Fibrinogen	*P*_trend_	GPIIb/IIIa	*P*_trend_	P-Selectin	*P*_trend_	TM	*P*_trend_	TPO	*P*_trend_
Be a healthy weight											
0	373 (16.5)	4.06 (3.85,4.27)	9.4 × 10^−8^	402 (362,446)	0.38	28.6 (25.5,32.0)	0.050	3.03 (2.8,3.3)	0.27	332 (304,363)	0.08
0.5	884 (39)	3.95 (3.76,4.15)		399 (361,442)		26.9 (24.1,30.1)		3.00 (2.8,3.2)		335 (308,365)	
1	1010 (44.5)	3.83 (3.65,4.03)		407 (368,451)		27.0 (24.2,30.2)		2.98 (2.8,3.2)		342 (314,373)	
Be physically active											
0	641 (28.3)	3.98 (3.79,4.19)	0.011	409 (369,453)	0.09	27.6 (24.7,30.8)	0.91	2.99 (2.8,3.2)	0.74	334 (306,364)	0.69
0.5	447 (19.7)	3.95 (3.75,4.16)		402 (363,446)		27.5 (24.5,30.7)		3.02 (2.8,3.2)		340 (311,370)	
1	1179 (52)	3.90 (3.71,4.10)		397 (359,439)		27.5 (24.6,30.7)		3.00 (2.8,3.2)		336 (309,366)	
Limit consumption of energy dense foods											
0	775 (34.2)	3.92 (3.73,4.13)	0.47	401 (362,444)	0.97	28.6 (25.6,32.0)	0.022	3.02 (2.8,3.2)	0.61	342 (314,373)	0.19
0.5	1323 (58.4)	3.95 (3.76,4.15)		393 (355,434)		28.1 (25.2,31.4)		3.02 (2.8,3.2)		337 (310,367)	
1	169 (7.4)	3.96 (3.75,4.18)		415 (372,464)		25.9 (23.0,29.2)		2.97 (2.7,3.2)		331 (301,363)	
Limit consumption of sugar sweetened drinks											
0	760 (33.5)	3.97 (3.77,4.18)	0.27	417 (377,462)	0.031	27.2 (24.4,30.4)	0.64	3.03 (2.8,3.3)	0.36	338 (310,368)	0.98
0.5	1408 (62.1)	3.93 (3.74,4.13)		409 (370,452)		27.3 (24.5,30.4)		2.99 (2.8,3.2)		340 (313,370)	
1	99 (4.4)	3.93 (3.71,4.16)		383 (341,429)		28.0 (24.7,31.8)		2.98 (2.8,3.2)		332 (301,366)	
Eat a diet rich in whole grains, vegetables, fruits, and beans											
Fruits and vegetables intake											
0	91 (4)	3.88 (3.65,4.12)	0.35	391 (347,442)	0.24	28.2 (24.7,32.2)	0.62	3.05 (2.8,3.3)	0.79	340 (307,377)	0.013
0.5	1621 (71.5)	3.96 (3.77,4.16)		395 (358,437)		27.5 (24.7,30.7)		2.99 (2.8,3.2)		332 (306,361)	
1	555 (24.5)	3.99 (3.80,4.20)		404 (365,447)		27.4 (24.5,30.6)		2.99 (2.8,3.2)		349 (321,380)	
Fiber intake											
0	237 (10.5)	3.94 (3.74,4.16)	0.80	412 (370,459)	0.43	26.8 (23.8,30.1)	0.06	2.99 (2.8,3.2)	0.24	325 (296,356)	0.09
0.5	1577 (69.7)	3.97 (3.78,4.17)		395 (359,437)		27.3 (24.5,30.4)		2.97 (2.8,3.2)		340 (313,370)	
1	453 (20.0)	3.96 (3.76,4.17)		398 (359,44)		28.6 (25.5,31.9)		3.06 (2.8,3.3)		343 (314,375)	
Limit consumption of red and processed meat											
0	1504 (66.3)	3.97 (3.79,4.17)	0.31	407 (369,449)	0.42	26.7 (24.0,29.7)	0.09	2.91 (2.7,3.1)	0.006	326 (300,354)	0.044
0.5	692 (30.6)	3.94 (3.75,4.14)		401 (363,443)		27.3 (24.5,30.5)		3.01 (2.8,3.2)		333 (306,362)	
1	71 (3.1)	3.92 (3.69,4.17)		400 (353,453)		28.6 (24.9,32.7)		3.09 (2.8,3.4)		351 (316,391)	
Limit alcohol consumption											
0	552 (24.3)	3.86 (3.66,4.06)	1.57 × 10^−7^	402 (362,445)	0.25	27.2 (24.3,30.4)	0.08	2.92 (2.7,3.1)	3.4 × 10^−7^	331 (304,361)	0.012
0.5	389 (17.2)	3.94 (3.74,4.15)		398 (358,441)		27.2 (24.3,30.5)		2.97 (2.8,3.2)		335 (307,366)	
1	1326 (58.5)	4.04 (3.85,4.25)		409 (370,452)		28.1 (25.2,31.3)		3.12 (2.9,3.3)		344 (316,374)	

Linear regression model adjusted for age, sex, education, smoking, aspirin intake, energy intake, CRP levels, LDL levels, prevalent cases of cancer, prevalent cases of myocardial infarction and stroke and each component of the score mutually, women only: menopausal status, use of hormone replacement therapy, use of contraceptive pills, full term pregnancy; GP = glycoprotein, TM = thrombomodulin; TPO = thrombopoietin; Plasma concentrations of fibrinogen in mg/mL, GPIIb/IIIa, P-Selectin and TM in ng/mL and TPO in pg/mL; EPIC = European Prospective Investigation into Cancer and Nutrition; WCRF/AICR = World Cancer Research Fund/American Institute for Cancer Research.

## Supplementary Materials

The following are available online at https://www.mdpi.com/2072-6643/11/9/2067/s1, Table S1: Geometric means (95% confidence intervals) of biomarker levels across WCRF/AICR categories within the EPIC-Heidelberg subcohort adjusted for age and sex (where applicable); Table S2: Multivariable adjusted geometric means (95% confidence intervals) of biomarker levels across WCRF/AICR categories within the EPIC-Heidelberg subcohort; Table S3: Geometric means (95% confidence intervals) of biomarker levels across single WCRF/AICR recommendations within men of the EPIC-Heidelberg subcohort; Table S4: Geometric means (95% confidence intervals) of biomarker levels across single WCRF/AICR recommendations within women of the EPIC-Heidelberg subcohort.

## Abbreviations

WCRF	World Cancer Research Fund;
AICR	American Institute of Cancer Research;
EPIC	European Prospective Investigation into Cancer and Nutrition;
GP	glycoprotein;
TM	thrombomodulin;
TPO	thrombopoietin;
CRP	C-reactive protein;
LDL	low density lipoprotein;
HDL	high density lipoprotein;
TG	triglycerides; BMI, body mass index;
ECLIA	electrochemiluminescence immunoassay;
ELISA	enzyme-linked immunosorbent assay;
QC	quality control;
CV	coefficient of variation;
Hb	hemoglobin;
CHD	coronary heart disease
