# Supplementary material for: Thrombomodulin and Thrombopoietin, Two Biomarkers of Hemostasis, Are Positively Associated with Adherence to the World Cancer Research Fund/American Institute for Cancer Research Recommendations for Cancer Prevention in a Population-Based Cross-Sectional Study
Source: Nutrients. 2019 Sep 3;11(9):2067. doi: 10.3390/nu11092067 (PMC6770787; doi:10.3390/nu11092067)
Supplement: Supplementary file 1 [file nutrients-11-02067-s001.pdf]

Supplementary Table 1: Geometric means (95% confidence intervals) of biomarker levels across WCRF/AICR categories within the EPIC-Heidelberg subcohort adjusted for age and sex (where applicable)

		WCRF/AICR score categories				
	**Subcohort**	**1**	**2**	**3**	**4**	P_{trend}
**All**						
n (%)	2267 (100)	373 (17)	805 (35)	748 (33)	341 (15)	
Score range	-	0 to ≤2/ 0 to ≤ 3	2 to ≤3/ 3 to ≤ 4	3 to ≤4/ 4 to ≤ 5	>4 / >5	
Fibrinogen (mg/ml)	3.99 (3.81,4.18)	3.86 (3.79,3.93)	3.85 (3.80,3.90)	3.77 (3.72,3.82)	3.69 (3.61,3.76)	<0.0001
Glycoprotein IIb/IIIa (ng/ml)	402 (367,441)	406 (392,420)	398 (388,407)	397 (387,406)	398 (383,412)	0.42
P-Selectin (ng/ml)	27.2 (24.6,30.0)	28.0 (27.0,29.1)	27.0 (26.4,27.8)	27.2 (26.5,28.0)	26.9 (25.9,28.0)	0.25
Thrombomodulin (ng/ml)	2.98 (2.8,3.2)	2.83 (2.8,2.9)	2.87 (2.8,2.9)	2.94 (2.9,3.0)	3.02 (2.9,3.1)	0.0001
Thrombopoietin (pg/ml)	337 (312,364)	333 (323,343)	335 (328,342)	346 (339,353)	352 (341,363)	0.001
**Men**						
n (%)	1071 (100)	158 (15)	421 (39)	367 (34)	125 (12)	
Score range	-	0 to ≤2	2 to ≤3	3 to ≤4	>4	
Fibrinogen (mg/ml)	3.94 (3.76,4.13)	3.91 (3.80,4.03)	3.83 (3.76,3.90)	3.76 (3.69,3.83)	3.72 (3.60,3.85)	0.01
Glycoprotein IIb/IIIa (ng/ml)	399 (364,438)	403 (382,426)	403 (390,417)	390 (377,405)	385 (362,409)	0.11
P-Selectin (ng/ml)	28.9 (26.2,32.0)	30.3 (28.5,32.3)	29.1 (28.0,30.2)	29.4 (28.2,30.6)	30.5 (28.4,32.7)	0.87
Thrombomodulin (ng/ml)	3.13 (2.9,3.3)	2.94 (2.8,3.0)	3.04 (3.0,3.1)	3.11 (3.0,3.2)	3.28 (3.2,3.4)	<0.0001
Thrombopoietin (pg/ml)	327 (302,354)	316 (302,330)	327 (318,336)	337 (327,347)	355 (338,373)	0.0002
**Women**						
n (%)	1196 (100)	215 (18)	384 (32)	381 (32)	216 (18)	
Score range	-	0 to ≤ 3	3 to ≤ 4	4 to ≤ 5	>5	
Fibrinogen (mg/ml)	4.04 (3.85,4.23)	3.83 (3.74,3.92)	3.88 (3.81,3.95)	3.79 (3.72,3.86)	3.67 (3.58,3.76)	0.002
GPIIb/IIIa (ng/ml)	405 (369,446)	408 (390,426)	392 (379,405)	403 (389,416)	406 (388,424)	0.75
P-Selectin (ng/ml)	25.5 (23.0,28.3)	25.9 (24.7,27.2)	25.2 (24.3,26.1)	25.2 (24.4,26.2)	24.2 (23.1,25.4)	0.07
Thrombomodulin (ng/ml)	2.84 (2.7,3.0)	2.72 (2.6,2.8)	2.71 (2.6,2.8)	2.77 (2.7,2.8)	2.81 (2.7,2.9)	0.10
Thrombopoietin (pg/ml)	347 (320,376)	349 (335,362)	344 (334,354)	355 (344,365)	355 (341,369)	0.25

Linear regression model adjusted for age and sex (where applicable); EPIC= European Prospective Investigation into Cancer and Nutrition; WCRF/AICR=World Cancer Research Fund/American Institute for Cancer Research.

Supplementary Table 2: Multivariable adjusted geometric means (95% confidence intervals) of biomarker levels across WCRF/AICR categories within the EPIC-Heidelberg subcohort

	Subcohort	WCRF/AICR score categories				
		1	2	3	4	P_{trend}
**All**						
n (%)	2267 (100)	373 (17)	805 (35)	748 (33)	341 (15)	
Score range	-	0 to ≤2/ 0 to ≤ 3	2 to ≤3/ 3 to ≤ 4	3 to ≤4/ 4 to ≤ 5	>4 / >5	
Fibrinogen (mg/ml)	3.99 (3.81,4.18)	3.88 (3.67,4.10)	3.90 (3.70,4.11)	3.86 (3.66,4.07)	3.85 (3.64,4.07)	0.39
Glycoprotein IIb/IIIa (ng/ml)	402 (367,441)	419 (375,469)	411 (369,458)	411 (368,458)	413 (369,462)	0.58
P-Selectin (ng/ml)	27.2 (24.6,30.0)	27.0 (24.1,30.2)	26.6 (23.9,29.6)	27.3 (24.5,30.4)	27.2 (24.3,30.5)	0.37
Thrombomodulin (ng/ml)	2.98 (2.8,3.2)	2.92 (2.7,3.2)	2.96 (2.7,3.2)	3.03 (2.8,3.3)	3.13 (2.9,3.4)	<0.0001
Thrombopoietin (pg/ml)	337 (312,364)	333 (302,366)	337 (307,369)	348 (317,382)	353 (321,389)	0.001
**Men**						
n (%)	1071 (100)	158 (15)	421 (39)	367 (34)	125 (12)	
Score range	-	0 to ≤2	2 to ≤3	3 to ≤4	>4	
Fibrinogen (mg/ml)	3.94 (3.76,4.13)	3.99 (3.71,4.29)	3.94 (3.68,4.22)	3.91 (3.65,4.19)	3.94 (3.66,4.24)	0.41
Glycoprotein IIb/IIIa (ng/ml)	399 (364,438)	396 (343,458)	397 (346,455)	382 (333,439)	378 (327,439)	0.12
P-Selectin (ng/ml)	28.9 (26.2,32.0)	30.9 (26.9,35.5)	30.1 (26.4,34.4)	31.7 (27.8,36.3)	32.5 (28.2,37.4)	0.057
Thrombomodulin (ng/ml)	3.13 (2.9,3.3)	3.00 (2.7,3.3)	3.11 (2.8,3.4)	3.18 (2.9,3.5)	3.37 (3.1,3.7)	<0.0001
Thrombopoietin (pg/ml)	327 (302,354)	295 (262,332)	310 (277,347)	320 (285,358)	339 (300,382)	0.0001
**Women**						
n (%)	1196 (100)	215 (18)	384 (32)	381 (32)	216 (18)	
Score range	-	0 to ≤ 3	3 to ≤ 4	4 to ≤ 5	>5	
Fibrinogen (mg/ml)	4.04 (3.85,4.23)	3.71 (3.31,4.16)	3.80 (3.40,4.25)	3.72 (3.33,4.16)	3.73 (3.32,4.18)	0.75
GPIIb/IIIa (ng/ml)	405 (369,446)	516 (406,656)	499 (394,632)	518 (409,657)	530 (416,677)	0.29
P-Selectin (ng/ml)	25.5 (23.0,28.3)	21.1 (16.6,26.6)	20.6 (16.3,25.9)	21.1 (16.7,26.6)	20.9 (16.5,26.5)	0.88
Thrombomodulin (ng/ml)	2.84 (2.7,3.0)	2.70 (2.3,3.2)	2.69 (2.3,3.2)	2.79 (2.4,3.3)	2.84 (2.4,3.4)	0.029
Thrombopoietin (pg/ml)	347 (320,376)	401 (327,491)	395 (323,482)	409 (335,501)	420 (342,517)	0.07

Linear regression model adjusted for age, sex (where applicable), education, smoking status, aspirin intake, energy intake, CRP level, LDL level, HbA1c, glucose, fasting status, prevalent cases of cancer, prevalent cases of myocardial infarction and stroke, women only: menopausal status, use of hormone replacement therapy, use of contraceptive pills, full term pregnancy; EPIC= European Prospective Investigation into Cancer and Nutrition; WCRF/AICR=World Cancer Research Fund/American Institute for Cancer Research.

Supplementary Table 3: Geometric means (95% confidence intervals) of biomarker levels across single WCRF/AICR recommendations within men of the EPIC-Heidelberg subcohort (n=1071)

Score	*n* (%)	Fibrinogen	P_{trend}	GPIIb/IIIa	P_{trend}	P-Selectin	P_{trend}	TM	P_{trend}	TPO	P_{trend}
Be a healthy weight											
0	184 (17.2)	4.19 (3.90,4.50)	0.0003	381 (331,440)	0.60	30.9 (26.3,36.3)	0.89	3.1 (2.8,3.4)	0.0156	305 (271,342)	0.0001
0.5	531 (49.6)	4.05 (3.79,4.34)		379 (331,433)		30.3 (26.0,35.3)		3.2 (2.9,3.5)		315 (282,351)	
1	356 (33.2)	3.94 (3.68,4.22)		386 (337,443)		30.9 (26.5,36.0)		3.2 (3.0,3.5)		336 (301,375)	
Be physically active											
0	287 (26.8)	4.12 (3.84,4.41)	0.26	388 (338,446)	0.47	30.7 (26.2,35.9)	0.90	3.1 (2.8,3.4)	0.08	313 (280,350)	0.54
0.5	210 (19.6)	4.01 (3.74,4.31)		378 (328,435)		30.6 (26.1,35.9)		3.2 (2.9,3.5)		323 (288,362)	
1	574 (53.6)	4.05 (3.78,4.33)		380 (332,436)		30.8 (26.4,35.9)		3.2 (2.9,3.5)		319 (285,356)	
Limit consumption of energy dense foods											
0	442 (41.3)	4.03 (3.76,4.32)	0.54	376 (328,431)	0.66	32.7 (28.0,38.2)	0.07	3.2 (2.9,3.5)	0.56	317 (284,355)	0.71
0.5	587 (54.8)	4.06 (3.79,4.35)		363 (317,415)		31.9 (27.3,37.2)		3.1 (2.9,3.4)		312 (280,349)	
1	42 (3.9)	4.09 (3.77,4.44)		409 (347,482)		27.8 (23.1,33.4)		3.2 (2.8,3.5)		325 (285,371)	
Limit consumption of sugary drinks											
0	397 (37.1)	4.08 (3.80,4.37)	0.67	406 (353,467)	0.006	30.8 (26.3,36.1)	0.69	3.3 (3.0,3.6)	0.08	312 (278,349)	0.25
0.5	623 (58.2)	4.06 (3.79,4.35)		388 (338,445)		30.2 (25.8,35.3)		3.2 (2.9,3.5)		317 (284,355)	
1	51 (4.8)	4.04 (3.74,4.37)		354 (304,413)		31.1 (26.1,37.0)		3.0 (2.7,3.4)		326 (288,369)	
Eat a diet rich in wholegrains, vegetables, fruits and beans											
Fruits and vegetables intake											
0	48 (4.5)	4.00 (3.68,4.35)	0.73	360 (305,425)	0.43	32.2 (26.7,38.8)	0.83	3.3 (2.9,3.6)	0.37	308 (270,352)	0.03
0.5	813 (75.9)	4.10 (3.84,4.38)		382 (334,436)		30.6 (26.3,35.5)		3.1 (2.9,3.4)		320 (287,356)	
1	210 (19.6)	4.05 (3.78,4.33)		385 (336,441)		30.8 (26.4,35.9)		3.1 (2.8,3.4)		335 (300,374)	
Fibre intake											
0	84 (7.8)	4.08 (3.77,4.41)	0.80	393 (337,458)	0.56	29.0 (24.4,34.5)	0.05	3.1 (2.8,3.5)	0.40	312 (275,353)	0.47
0.5	726 (67.8)	4.08 (3.82,4.36)		382 (335,436)		30.3 (26.1,35.2)		3.10 (2.8,3.4)		326 (293,363)	
1	261 (24.4)	4.06 (3.79,4.35)		379 (331,435)		32.2 (27.6,37.6)		3.18 (2.9,3.5)		326 (292,364)	

Supplementary Table 3 continues

Supplementary Table 3 continued

Limit consumption of red and processed meat										
0	926 (89.8)	4.02 (3.77,4.29)	0.60	398 (350,453)	0.09	29.2 (25.2,33.7)	0.06	3.01 (2.8,3.3)	0.006	317 (286,352)	0.88
0.5	86 (8)	4.03 (3.75,4.33)		367 (318,423)		32.5 (27.7,38.2)		3.24 (3.0,3.6)		308 (275,346)	
1	23 (2.1)	4.13 (3.75,4.55)		382 (316,462)		30.5 (24.5,37.9)		3.24 (2.9,3.7)		330 (282,385)	
Limit alcohol consumption											
0	340 (31.7)	3.97 (3.71,4.26)	0.007	385 (335,442)	0.83	30.4 (26.0,35.6)	0.19	3.11 (2.8,3.4)	0.0004	310 (278,347)	0.006
0.5	185 (17.3)	4.08 (3.80,4.39)		379 (329,437)		30.1 (25.6,35.4)		3.08 (2.8,3.4)		317 (282,355)	
1	546 (51.1)	4.12 (3.85,4.41)		382 (334,438)		31.5 (27.1,36.8)		3.30 (3.0,3.6)		328 (294,366)	

Linear regression model adjusted for age, education, smoking, aspirin intake, energy intake, CRP levels, LDL levels, prevalent cases of cancer, prevalent cases of myocardial infarction and stroke and each component of the score mutually; GP= Glycoprotein, TM= Thrombomodulin; TPO=Thrombopoietin; Plasma concentrations of fibrinogen in mg/ml, GPIIb/IIIa, P-Selectin and TM in ng/ml and TPO in pg/ml; EPIC= European Prospective Investigation into Cancer and Nutrition; WCRF/AICR=World Cancer Research Fund/American Institute for Cancer Research.

Supplementary Table 4: Geometric means (95% confidence intervals) of biomarker levels across single WCRF/AICR recommendations within women of the EPIC-Heidelberg subcohort (n=1196)

Score	*n* (%)	Fibrinogen	P_{trend}	GPIIb/IIIa	P_{trend}	P-Selectin	P_{trend}	TM	P_{trend}	TPO	P_{trend}
Be a healthy weight											
0	189 (15.8)	3.81 (3.48,4.18)	0.002	440 (363,531)	0.38	21.0 (17.3,25.6)	0.024	2.82 (2.4,3.2)	0.005	343 (291,406)	0.78
0.5	353 (29.5)	3.77 (3.44,4.13)		436 (361,527)		19.3 (15.8,23.5)		2.69 (2.3,3.1)		346 (293,409)	
1	654 (54.7)	3.66 (3.34,4.01)		448 (371,540)		19.3 (15.9,23.4)		2.64 (2.3,3.0)		342 (291,403)	
Be physically active											
0	354 (29.6)	3.78 (3.45,4.14)	0.004	448 (371,540)	0.10	20.0 (16.4,24.3)	0.72	2.72 (2.4,3.1)	0.21	343 (291,404)	0.87
0.5	237 (19.8)	3.79 (3.46,4.16)		444 (367,537)		19.8 (16.2,24.1)		2.75 (2.4,3.2)		345 (292,407)	
1	605 (50.6)	3.67 (3.35,4.02)		432 (358,521)		19.8 (16.3,24.1)		2.67 (2.3,3.1)		344 (292,406)	
Limit consumption of energy dense foods											
0	333 (27.8)	3.74 (3.41,4.10)	0.76	438 (362,529)	0.68	20.4 (16.8,24.9)	0.16	2.70 (2.3,3.1)	0.82	350 (297,413)	0.28
0.5	736 (61.5)	3.75 (3.43,4.11)		436 (362,526)		20.3 (16.7,24.6)		2.75 (2.4,3.2)		348 (296,410)	
1	127 (10.6)	3.75 (3.41,4.12)		449 (370,545)		19.0 (15.5,23.1)		2.68 (2.3,3.1)		334 (282,396)	
Limit consumption of sugary drinks											
0	390 (30.4)	3.80 (3.47,4.16)	0.12	445 (369,537)	0.83	19.5 (16.1,23.7)	0.37	2.68 (2.3,3.1)	0.75	355 (301,418)	0.26
0.5	843 (65.6)	3.74 (3.42,4.10)		447 (372,537)		19.9 (16.4,24.1)		2.66 (2.3,3.1)		354 (301,416)	
1	52 (4)	3.70 (3.35,4.09)		431 (351,529)		20.1 (16.3,24.9)		2.79 (2.4,3.3)		324 (271,388)	
Eat a diet rich in wholegrains, vegetables, fruits and beans											
Fruits and vegetables intake											
0	43 (3.6)	3.85 (3.49,4.25)	0.03	422 (346,516)	0.33	23.7 (19.2,29.2)	0.63	2.95 (2.5,3.4)	0.80	398 (334,474)	0.12
0.5	808 (67.6)	3.90 (3.58,4.26)		402 (337,480)		24.0 (19.9,28.9)		2.97 (2.6,3.4)		368 (3150,430)	
1	345 (28.8)	4.00 (3.67,4.37)		416 (348,498)		23.6 (19.6,28.5)		2.98 (2.6,3.4)		387 (331,453)	
Fibre intake											
0	153 (12.8)	3.92 (3.58,4.29)	0.65	428 (356,514)	0.45	23.5 (19.4,28.5)	0.50	2.99 (2.6,3.4)	0.72	363 (309,427)	0.15
0.5	851 (71.2)	3.93 (3.60,4.29)		403 (338,481)		23.8 (19.7,28.6)		2.96 (2.6,3.4)		382 (327,447)	
1	192 (16.1)	3.96 (3.62,4.33)		412 (343,495)		24.3 (20.0,29.5)		3.02 (2.6,3.5)		386 (329,454)	

Supplementary Table 4 continues

Supplementary Table 4 continued

Limit consumption of red and processed meat											
0	542 (45.3)	3.80 (3.47,4.16)	0.17	441 (366,531)	0.94	19.4 (16.0,23.6)	0.45	2.65 (2.3,3.1)	0.08	332 (282,390)	0.047
0.5	606 (50.7)	3.76 (3.43,4.11)		442 (367,532)		19.6 (16.1,23.7)		2.73 (2.4,3.1)		342 (291,402)	
1	48 (4)	3.69 (3.33,4.08)		440 (358,541)		20.6 (16.6,25.5)		2.75 (2.4,3.2)		359 (299,430)	
Limit alcohol consumption											
0	212 (17.7)	3.65 (3.32,4.00)	$<3.5*10^{-6}$	433 (358,524)	0.04	19.7 (16.1,24.0)	0.46	2.60 (2.3,3.0)	0.0003	344 (291,407)	0.92
0.5	204 (17.1)	3.73 (3.40,4.09)		435 (360,526)		19.9 (16.3,24.2)		2.74 (2.4,3.2)		343 (290,404)	
1	780 (65.2)	3.87 (3.53,4.23)		455 (378,548)		20.1 (16.5,24.3)		2.80 (2.4,3.2)		345 (293,406)	
Breastfeed your baby, if you can											
0	390 (32.6)	3.76 (3.44,4.12)	0.10	447 (371,539)	0.09	19.8 (16.3,24.0)	0.77	2.70 (2.4,3.1)	0.92	354 (301,417)	0.12
0.5	497 (41.6)	3.80 (3.46,4.16)		450 (372,544)		19.8 (16.3,24.1)		2.72 (2.4,3.1)		340 (288,402)	
1	309 (25.8)	3.68 (3.36,4.04)		426 (352,516)		20.0 (16.4,24.4)		2.71 (2.4,3.1)		337 (285,399)	

Linear regression model adjusted for age, education, smoking, aspirin intake, energy intake, CRP levels, LDL levels, prevalent cases of cancer, prevalent cases of myocardial infarction and stroke, menopausal status, use of hormone replacement therapy, use of contraceptive pills, full term pregnancy and each component of the score mutually; GP= Glycoprotein, TM= Thrombomodulin; TPO=Thrombopoietin; Plasma concentrations of fibrinogen in mg/ml, GPIIb/IIIa, P-Selectin and TM in ng/ml and TPO in pg/ml; EPIC= European Prospective Investigation into Cancer and Nutrition; WCRF/AICR=World Cancer Research Fund/American Institute for Cancer Research.
